# Association of Visceral Obesity-Related Indices With Coronary Collateralization in Patients With Chronic Total Occlusion

**DOI:** 10.3389/fcvm.2021.742855

**Published:** 2021-10-21

**Authors:** Meng-Jiao Shao, Jun-yi Luo, Jia Shi, Fen Liu, Chun-fang Shan, Fan Luo, Xiao-lin Yu, Qian Zhao, Ting Tian, Xiao-Mei Li, Yi-ning Yang

**Affiliations:** ^1^Department of Cardiology, The First Affiliated Hospital of Xinjiang Medical University, Urumqi, China; ^2^Xinjiang Key Laboratory of Cardiovascular Disease Research, Clinical Medical Research Institute of First Affiliated Hospital of Xinjiang Medical University, Urumqi, China; ^3^Department of Cardiology, People's Hospital of Xinjiang Uygur Autonomous Region, Urumqi, China

**Keywords:** chronic total occlusion (CTO), coronary collateralization, obesity, visceral obesity-related indices, Chinese visceral adiposity index

## Abstract

**Background:** Obesity is an independent risk factor for cardiovascular disease. We investigated whether and to what extent visceral obesity-related indices were associated with coronary collateralization (CC) in chronic total occlusion (CTO) patients.

**Methods:** This retrospective cohort study involved 1,008 consecutive patients with CTO who underwent CTO-percutaneous coronary artery intervention (PCI). CC was graded according to the Rentrop scoring system. Data on demographic and clinical characteristics were collected by cardiovascular doctors. Logistic regression, receiver operating characteristic (ROC) curve and Kaplan-Meier analyses were performed to assess the predictive value of visceral obesity-related indices for CC.

**Results:** Overall, 1,008 inpatients were assigned to the poor CC group (*n* = 592) and good CC group (*n* = 416). In multivariate-adjusted logistic regression analyses, all visceral obesity-related indices (*P*-value < 0.001) were significantly associated with CC. After ROC analysis and the *Delong* test, the Chinese visceral adiposity index (CVAI) had the largest area under the curve (AUC) of 0.741 (0.711–0.771). Further analysis revealed that CVAI quartile remained a risk factor for poor CC in all groups, CVAI was associated with a 1.018-fold higher risk of poor CC (OR = 1.018, 95% CI: 1.014–1.021, *P* < 0.001). Individuals in the top CVAI quartile group had the highest risk of poor CC (OR = 10.657, 95% CI: 6.492–17.493, *P* < 0.001). Subgroup analyses showed similar results, and CVAI quartile remained a risk factor for poor CC. Moreover, increased CVAI predicted poor prognosis in CTO patients.

**Conclusion:** In summary, this study indicated that all the increased visceral obesity-related indices were significantly associated with increased poor CC risk. After adjusting for potential risks, CVAI had the best performance for estimating CC and predicting prognosis in CTO patients.

## Introduction

Chronic coronary total occlusions (CTOs) are defined as 100% occlusions with Thrombolysis in Myocardial Infarction (TIMI) 0 flow and at least a 3-month duration (1). Coronary collateralization (CC) is a process that utilizes a network of arterial-arterial anastomotic connections present in the heart between vascular branches from different regions to provide an alternative source of blood supply to the myocardium jeopardized by ischemia (2–4). Good CC contributes to a reduction in infarct size, relieves angina, protects cardiac function, and reduces mortality risk (5–7). In addition, good CC increases the likelihood of successful CTO percutaneous coronary intervention (PCI) (8).

Additionally, there is growing evidence showing that obesity is associated with cardiovascular disease (CVD) risk (9). Previous studies have demonstrated the correlation between body mass index (BMI), the most widely used clinical indicator for evaluating obesity, and CC and have shown that compared to patients with a normal BMI, obese patients (defined as BMI ≥ 30 kg/m^2^) have poor CC and a significantly higher risk of major adverse cardiovascular events (10). Moreover, existing obesity-related indices such as BMI evaluate systemic obesity, but individuals with the same BMI may have different amounts of body fat and visceral fat (11–13). Therefore, the concept of novel visceral obesity-related indices, such as the waist-to-height ratio (WHtR), lipid accumulation product (LAP), cardiometabolic index (CMI), body adiposity index (BAI) and Chinese visceral adiposity index (CVAI), was developed in this context. Moreover, an increasing number of studies have reported that visceral obesity, compared to overall obesity, might be a better determinant of cardiovascular disease (CVD) risk (14, 15). To the best of our knowledge, there has been no study evaluating the correlations of various visceral obesity-related indices with CC of patients with CTO. Thus, we determined the association of visceral obesity-related indices with CC of patients with CTO.

## Methods

The protocol was approved by the Human Ethics Committee of the First Affiliated Hospital of Xinjiang Medical University (Approval ID: K202102-17) and complied with the standards of the Declaration of Helsinki. All patients gave written informed consent.

### Study Design and Population

This was a retrospective cohort study that recruited 2,318 consecutive patients with CTO who underwent CTO-PCI between January 2017 and July 2020 at the First Affiliated Hospital of Xinjiang Medical University.

**Figure 1:**
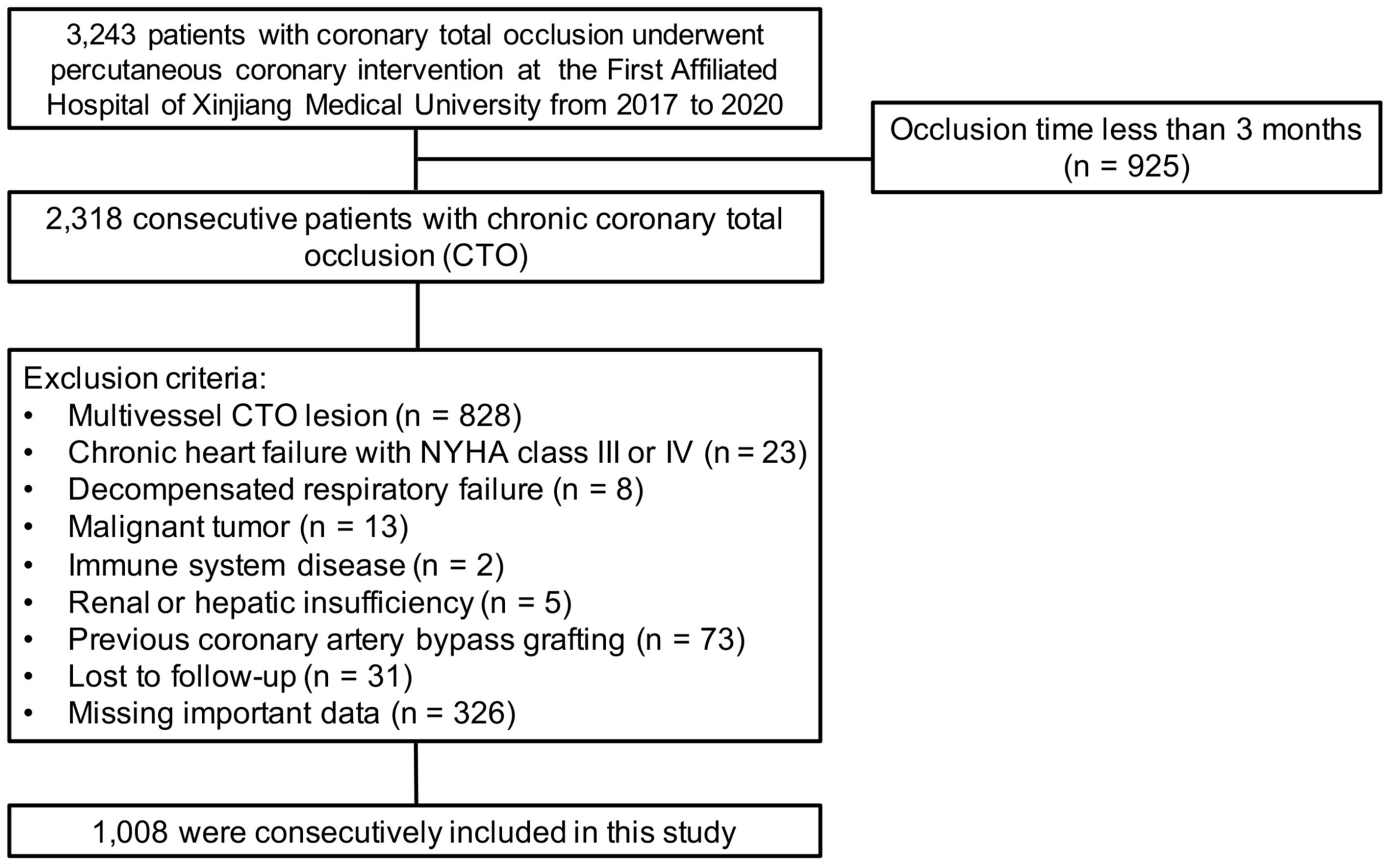
Flow chart of patient enrollment.

The exclusion criteria were occlusion time <3 months, chronic heart failure with New York Heart Association (NYHA) class III or IV, decompensated respiratory failure, malignant tumor, immune system disease, renal or hepatic insufficiency, severe valvular disease, and previous coronary artery bypass grafting surgery, as these conditions might influence CC formation. We also excluded an additional 31 patients who were lost to follow-up, 326 patients who were missing important data and 828 patients who had multivessel CTO lesions. Finally, the remaining 1,008 patients were enrolled in the analyses. The detailed recruitment process is depicted in Figure 1.

### Baseline Data Collection and Definitions

Demographic information collected at baseline included sex, age, medical history, and smoking status. Physical examination included the measurement of height, weight, waist circumference (WC), blood pressure, resting heart rate (RHR) and abdominal ultrasonography. BMI was calculated as weight (in kilograms) divided by height (in meters) squared. WC was measured 1 cm above the umbilical level after the participant had exhaled. Hip circumference was measured at the level of maximum extension of the hip. Blood samples were drawn from all participants and analyzed by an automated biochemical analyzer; blood indices included creatinine, lipid profiles, glucose, and glycated albumin.

The visceral obesity-related indices used in this study are listed as follows:

Waist-hip ratio (WHR) was calculated as WC divided by hip circumference (cm/cm).
Waist-to-height ratio (WHtR) was calculated as WC divided by height (cm/cm).
Body adiposity index (BAI) (16) was calculated as [Hip circumference (cm)/Height (cm)^1.5^-18].

Chinese visceral adiposity index (CVAI) (17) was calculated as follows:

CVAI in men: −267.93 + 0.68 × age + 0.03 × BMI (kg/m^2^) + 4.00 × WC (cm) + 22.00 × Log_10_ triglycerides (TGs) (mmol/L) – 16.32 × HDL-C (mmol/L)
CVAI in women: −187.32 + 1.71 × age + 4.23 × BMI (kg/m^2^) + 1.12 × WC (cm) + 39.76 × Log_10_TGs (mmol/L) – 11.66 × HDL-C (mmol/L)

Lipid accumulation product (LAP) (18) was calculated as [WC (cm) – 65] × [TG (mmol/L)] in men and [WC (cm) – 58] × [TG (mmol/L)] in women.

Cardiometabolic index (CMI) (19) was calculated as WHtR × [TG (mmol/L)/HDL cholesterol (mmol/L)].

### PCI, Coronary Collateral Scoring, and Study Endpoint

The PCI procedure was performed as previously described (20, 21). Coronary angiography and the intervention were performed with standard techniques using 6F or 7F guiding catheters *via* the radial or femoral approach and drug eluting stent implantation as the default strategy. Patients were given aspirin (300 mg) + clopidogrel (300 mg) or ticagrelor (180 mg) orally before the operation and were given secondary clinical prophylactic medication after PCI. After discharge, all patients were encouraged to take guideline-recommended medications, including statins, angiotensin-converting enzyme inhibitors and β-blockers, unless contraindicated.

Coronary collateralization was visually estimated using the Rentrop standard (22). Good CC was defined as Rentrop 2 or 3, and poor CC was defined as Rentrop 0 or 1. All angiograms were viewed by more than two experienced interventional physicians. Any difference in interpretation was resolved by a third reviewer.

The study endpoint was the occurrence of composite major adverse cardio-cerebral events (MACCEs) during follow-up, including all-cause death, cardiac death, angina pectoris readmission, non-fatal myocardial infarction, malignant arrhythmia, severe cardiac insufficiency (NYHA class III or IV), stent restenosis, target vessel revascularization and non-fatal stroke.

### Statistical Analysis

Continuous data are presented as the means ± standard deviations (SDs) and were compared by Student's *t*-test. Categorical data are presented as percentages of the total in each category and were compared by Pearson chi-square test. The comparison of measurement data among quartiles of CVAI groups and Rentrop classification groups was based on one-way ANOVA. Univariable and multivariable logistic regression analyses were executed to observe the relationship between CC and visceral obesity-related indices. In multivariate analysis, odds ratios (ORs) and 95% confidence intervals (CIs) for CC were calculated using the logistic regression model after adjusting for potential confounding variables. Receiver operating characteristic (ROC) curves were constructed, and the areas under the curves (AUCs) were used to estimate the ability of each visceral obesity-related index to predict CC. Moreover, comparisons of AUC values were performed using the *DeLong* test, and the optimal cutoff value was identified using the maximum value of Youden's index. Kaplan-Meier analysis was used to compare the probability of MACCEs in CTO patients based on the *DeLong* test of the ROC curve. Then, *Bonferroni* multiple-test correction was applied to the obtained *P*-values.

All statistical analyses were performed using SPSS version 26.0 (SPSS Inc., Chicago, IL, USA) and R software (version 3.6.3), with the pROC, survival and survminer packages. All tests were two-sided, and a *P*-value < 0.05 was considered statistically significant.

## Results

### Baseline Characteristics

A total of 1,008 patients [735 (72.9%) men, mean age 60.89 ± 9.95 years] were included in our study. There were 592 (58.7%) patients with poor CC and 416 (41.3%) with good CC. Compared to patients with good CC, there were no differences in poor CC from the ethnic groups. Compared to patients with good CC, the prevalence of T2DM, age and sex were significantly different among the CTO patients with poor CC (all *P* < 0.001). There were no significant differences in the incidence of hypertension or stroke between the patients with poor and good CC. Compared to patients with good CC, visceral obesity-related indices, including BMI, WHR, WtHR, CMI, LAP, BAI, and CVAI, were higher in poor-CC patients (all *P* < 0.001). The RHR, TG, NT-proBNP and fasting blood glucose (FBG) levels were more elevated in patients with poor CC (all *P* < 0.0.05; Table 1).

**Table 1:** Clinical baseline characteristics and obesity-related indices of the patients with CTO.

**Variable**	**All patients (*n* = 1,008)**	**Poor CCs (*n* = 592)**	**Good CCs (*n* = 416)**	***p-*value**
Age (years)	60.89 ± 9.95	62.47 ± 9.88	58.63 ± 9.16	<0.001
Men (%)	735 (72.9)	412 (69.6)	323 (77.6)	0.005
Ethnicity (%)
Hans	579 (57.4)	347 (58.6)	232 (55.8)	0.569
Uyghurs	328 (32.6)	192 (32.4)	136 (32.7)	
Kazakhs	77 (7.6)	40 (6.8)	37 (8.9)	
Other ethnicities	24 (2.4)	13 (2.2)	11 (2.6)	
Hypertension (%)	682 (67.7)	400 (67.6)	282 (67.8)	0.498
T2DM (%)	424 (42.1)	279 (47.1)	145 (34.9)	<0.001
Current smoker (%)	358 (35.5)	218 (36.8)	140 (33.7)	0.316
Stroke/TIA (%)	114 (11.3)	70 (11.8)	44 (10.6)	0.614
Obesity-related indices
BMI (kg/m^2^)	27.84 ± 4.61	28.82 ± 5.02	26.59 ± 3.62	<0.001
Waist-to-hip ratio	0.96 ± 0.07	0.97 ± 0.08	0.94 ± 0.06	<0.001
Waist-to-height ratio	0.58 ± 0.08	0.60 ± 0.09	0.56 ± 0.07	<0.001
Cardiometabolic index	1.48 ± 1.08	1.65 ± 1.19	1.25 ± 0.87	<0.001
Lipid accumulation product	76.71 ± 60.994	90.28 ± 68.27	57.93 ± 41.85	<0.001
Body adiposity index	28.89 ± 6.01	29.57 ± 6.14	27.93 ± 5.67	<0.001
Chinese visceral adiposity index	149.92 ± 56.77	125.32 ± 46.86	81.43 ± 46.28	<0.001
FBG (mmol/L)	6.58 ± 3.46	6.85 ± 4.08	6.21± 2.28	0.004
HbA1c (%)	6.83 ± 1.51	7.05 ± 1.60	6.53 ± 1.34	<0.001
HBP (mmHg)	129.18 ± 19.05	130.01 ± 20.06	128.01 ± 17.48	0.102
SBP (mmHg)	75.90 ± 12.89	75.88 ± 12.94	75.92 ± 12.87	0.955
RHR (bpm)	77.45 ± 1.21	78.36± 10.65	76.15 ± 11.86	0.002
Total cholesterol (mmol/l)	3.73 ±1.04	3.74 ± 1.00	3.72 ± 1.06	0.726
Triglycerides (mmol/L)	1.84 ± 1.30	2.03 ± 1.37	1.58 ± 1.18	<0.001
LDL cholesterol (mmol/l)	2.32 ± 0.90	2.33 ± 0.89	2.31 ± 0.89	0.786
HDL cholesterol (mmol/l)	1.01 ± 0.28	0.99 ± 0.25	1.02 ± 0.30	0.134
eGFR (ml/min/1.73 m^2^)	95.78 ± 15.85	95.20 ± 16.31	96.61 ± 15.17	0.201
NT-proBNP (pg/ml)	347.74 ± 743.18	421.98 ± 878.12	241.98 ± 466.84	<0.001
Echocardiography
LA (mm)	36.61 ± 4.77	37.14 ± 5.13	35.86 ± 4.09	0.001
LVEDD (mm)	51.84 ± 14.29	53.11 ± 18.14	50.05 ± 4.96	<0.001
LVESD (mm)	35.25 ± 6.45	36.38 ± 7.32	33.68 ± 7.323	<0.001
LVEF (%)	59.26 ± 7.48	57.87 ± 8.43	61.19 ± 5.33	<0.001
Location of CTO lesion (%)
Left anterior descend	379 (37.6)	234 (39.5)	145 (34.9)	0.291
Left circumflex artery	226 (22.4)	126 (21.3)	100 (24.0)	
Right coronary artery	403 (40.0)	232 (39.2)	171 (41.1)	
Medications
Oral antiplatelet agent	1,008 (100)	584 (100)	424 (100)	1.000
Statin	983 (97.8)	578 (98.0)	405 (97.6)	0.827
β-blocker	621 (61.8)	382 (64.7)	23.9 (57.6)	0.025
ACEI or ARB	426 (42.4)	278 (47.2)	148 (35.7)	0.004

*Data are expressed as mean ± standard deviation or number*.

*CTO, chronic total occlusion; CCs, coronary collateralizations; T2DM, type 2 diabetes mellitus; SBP, systolic blood pressure; DBP, diastolic blood pressure; FPB, fasting plasma glucose; HbA1c, glycosylated hemoglobin; HDL, high-density lipoprotein; LDL, low-density lipoprotein; LA, left atrium; RA, Right atrium; LVEDD, left ventricular end diastolic diameter; LVESD, left ventricular end systolic diameter; LVEF, left ventricular ejection fraction; ACEI, angiotensin-converting enzyme inhibitor; ARB, angiotensin receptor blocker; RHR, Resting heart rate; TIA, Transient ischemic attack; eGFR, estimated Glomerular filtration rate*.

### Visceral Obesity-Related Indices With CC

#### Elevated Visceral Obesity-Related Indices Are Independent Risk Factors for Poor CC

We used univariate and multivariate logistic regression models to analyze the visceral obesity-related indices, including BMI, WHR, WHtR, CMI, LAP and CVAI, with both continuous and categorical levels, that were strongly associated with CC after adjustment for potential confounders.

After adjustment for several potential risk factors, such as age, sex, CTO duration, RHR, current smoking status, history of T2DM, FBG, TGs, left ventricular ejection fraction (LVEF), quartiles of BMI (adjusted OR = 1.106, 95% CI 1.067–1.146, *P* < 0.001), WHR (adjusted OR = 1.106, 95% CI 1.080–1.133, *P* < 0.001), WHtR (adjusted OR = 1.065, 95% CI 1.045–1.085, *P* < 0.001), CMI (adjusted OR = 1.349, 95% CI 1.011–1.799, *P* < 0.001), LAP (adjusted OR = 1.034, 95% CI 1.018–1.051, *P* < 0.001), BAI (adjusted OR = 1.038, 95% CI 1.013–1.065, *P* < 0.001), and CVAI (adjusted OR = 1.018, 95% CI 1.014–1.021, *P* < 0.001) remained independent predictors of poor CC. Compared to the first quartile, the top quartile group had the highest ORs of incident poor CC, BMI [2.793 (1.806–4.317), *P* < 0.001], WHR [5.106 (3.228–8.078), *P* < 0.001], WtHR [3.822 (2.440–5.985), *P* < 0.001], CMI [2.666 (1.781–3.990), *P* < 0.001], LAP [7.179 (3.891–13.247), *P* < 0.001], BAI [1.906 (1.256–2.893), *P* = 0.002], and CVAI [10.657 (6.492–17.493), *P* < 0.001], after adjusting for potential risk factors (Table 2).

**Table 2:** Impact of obesity-related indices on coronary collateral growth in patients with CTO.

	**N (%)**	**Poor/good**	**Model 1**		**Model 2**		**Model 3**		**Model 4**	
			**OR (95%CI)**	***P*-value**	**Adjusted OR (95%CI)**	***P-*value**	**Adjusted OR (95%CI)**	***P-*value**	**Adjusted OR (95%CI)**	***P-*value**
BMI (kg/m^2^) (continuous)	1,008 (100)	592/416	1.117 (1.083–1.152)	<0.001	1.126 (1.091–1.163)	<0.001	1.127 (1.089–1.167)	<0.001	1.106 (1.067–1.146)	<0.001
BMI quartiles										
Q1 ≤ 24.61	254 (25.2)	119/135	Ref.		Ref.		Ref.		Ref.	
24.61 < Q2 ≤ 26.83	254 (25.2)	140/114	1.393 (0.983–1.975)	0.063	1.458 (1.015–2.095)	0.042	1.491 (1.017–2.186)	0.041	1.312 (0.882–1.951)	0.18
26.83 < Q3 ≤ 30.09	249 (24.7)	144/105	1.556 (1.094–23,213)	0.014	1.566 (1.086–2.259)	0.016	1.667 (1.139–2.440)	0.009	1.353 (0.908–2.017)	0.138
Q4 > 30.09	251 (24.9)	189/62	3.458 (2.369–5.048)	<0.001	3.696 (2.486–5.496)	<0.001	3.591 (2.356–5.442)	<0.001	2.793 (1.806–4.317)	<0.001
WHR (continuous, per 100 unit)	1,008 (100)	592/416	1.104 (1.081–1.128)	<0.001	1.103 (1.079–1.127)	<0.001	1.106 (1.081–1.132)	<0.001	1.106 (1.080–1.133)	<0.001
WHR quartiles										
Q1 ≤ 91.00	276 (27.4)	118/158	Ref.		Ref.		Ref.		Ref.	
91.00 < Q2 ≤ 94.29	229 (22.7)	116/113	1.375 (0.967–1.955)	0.077	1.227 (0.851–1.770)	0.273	1.162 (0.791–1.707)	0.443	0.999 (0.663–1.505)	0.996
94.29 < Q3 ≤ 101.00	275 (27.3)	171/104	2.202 (1.565–3.096)	<0.001	2.041 (1.434–2.906)	<0.001	2.026 (1.395–2.941)	<0.001	1.980 (1.339–2.929)	0.001
Q4 > 101.00	228 (22.6)	187/41	6.107 (4.038–9.236)	<0.001	5.841 (3.813–8.947)	<0.001	5.607 (3.591–8.756)	<0.001	5.106 (3.228–8.078)	<0.001
WHtR (continuous, per 100 unit)	1,008 (100)	592/416	1.068 (1.050–1.086)	<0.001	1.069 (1.051–1.088)	<0.001	1.069 (1.049–1.088)	<0.001	1.065 (1.045–1.085)	<0.001
WHtR quartiles										
Q1 ≤ 52.00	279 (27.7)	132/147	Ref.		Ref.		Ref.		Ref.	
52.00 < Q2 ≤ 57.50	225 (22.3)	104/121	0.957 (0.673–1.361)	0.807	0.949 (0.658–1.369)	0.78	0.863 (0.587–1.268)	0.453	0.731 (0.487–1.098)	0.131
57.50 < Q3 ≤ 64.00	269 (26.7)	168/101	1.852 (1.317–2.605)	<0.001	1.781 (1.251–2.353)	0.001	1.629 (1.124–2.359)	0.01	1.598 (1.082–2.358)	0.018
Q4 > 64.00	235 (23.3)	188/47	4.455 (2.996–6.624)	<0.001	4.440 (2.941–6.702)	<0.001	4.266 (2.775–6.556)	<0.001	3.822 (2.440–5.985)	<0.001
CMI (continuous)	1,008 (100)	592/416	1.491 (1.294–1.719)	<0.001	1.597 (1.372–1.859)	<0.001	1.600 (1.364–1.872)	<0.001	1.349 (1.011–1.799)	<0.001
CMI quartiles										
Q1 ≤ 0.73	262 (26.0)	131/131	Ref.		Ref.		Ref.		Ref.	
0.73 < Q2 ≤ 1.18	247 (24.5)	122/125	0.976 (0.686–1.382)	0.891	1.023 (0.709–1.477)	0.901	1.000 (0.682–1.467)	0.987	0.872 (0.577–1.319)	0.518
1.18 < Q3 ≤ 1.90	247 (24.5)	168/79	2.127 (1.483–3.050)	<0.001	2.556 (1.741–3.753)	<0.001	2.705 (1.800–4.067)	<0.001	1.994 (1.200–3.314)	0.008
Q4 > 1.90	252 (25.0)	171/81	2.111 (1.475–3.021)	<0.001	2.635 (1.799–3.859)	<0.001	2.477 (1.726–3.005)	<0.001	2.666 (1.781–3.990)	<0.001
LAP (continuous)	1,008 (100)	592/416	1.012 (1.009–1.015)	<0.001	1.013 (1.010–1.016)	<0.001	1.013 (1.010–1.017)	<0.001	1.034 (1.018–1.051)	<0.001
LAP quartiles										
Q1 ≤ 35.70	253 (25.1)	103/150	Ref.		Ref.		Ref.		Ref.	
35.70 < Q2 ≤ 58.99	251 (24.9)	140/111	1.837 (1.290–2.616)	0.001	1.968 (1.359–2.851)	<0.001	1.960 (1.332–2.883)	0.001	1.829 (1.220–2.740)	0.003
58.99 < Q3 ≤ 93.78	252 (25.0)	149/103	2.107 (1.477–3.004)	<0.001	2.529 (1.735–3.678)	<0.001	2.794 (1.872–4.171)	<0.001	2.619 (1.676–4.095)	<0.001
Q4 > 93.78	252 (25.0)	200/52	5.601 (3.774–8.314)	<0.001	6.524 (4.308–9.880)	<0.001	6.605 (4.271–10.214)	<0.001	7.179 (3.891–13.247)	<0.001
BAI (continuous)	1,008 (100)	592/416	1.048 (1.025–1.071)	<0.001	1.048 (1.025–1.072)	<0.001	1.043 (1.019–1.068)	<0.001	1.038 (1.013–1.065)	<0.001
BAI quartiles										
Q1 ≤ 24.56	256 (25.4)	127/129	Ref.		Ref.		Ref.		Ref.	
24.56 < Q2 ≤ 28.56	249 (24.7)	144/105	1.393 (0.981–1.979)	0.064	1.385 (0.962–1.993)	0.08	1.316 (0.900–1.925)	0.157	1.272 (0.856–1.890)	0.234
28.56 < Q3 ≤ 32.52	253 (25.1)	149/104	1.455 (1.025–2.066)	0.036	1.331 (0.924–1.918)	0.124	1.253 (0.856–1.833)	0.246	1.310 (0.879–1.952)	0.184
Q4 > 32.52	250 (24.8)	172/78	2.240 (1.558–3.219)	<0.001	2.292 (1.571–3.343)	<0.001	2.163 (1.455–3.216)	<0.001	1.906 (1.256–2.893)	0.002
CVAI (continuous)	1,008 (100)	592/416	1.019 (1.016–1.023)	<0.001	1.019 (1.016–1.022)	<0.001	1.019 (1.016–1.022)	<0.001	1.018 (1.014–1.021)	<0.001
CVAI quartiles										
Q1 ≤ 71.41	252 (25.0)	83/169	Ref.		Ref.		Ref.		Ref.	
71.41 < Q2 ≤ 106.67	252 (25.0)	131/121	2.204 (1.537–3.162)	<0.001	2.104 (1.448–3.056)	<0.001	2.149 (1.447–3.190)	<0.001	2.169 (1.436–3.278)	<0.001
106.67 < Q3 ≤ 143.64	252 (25.0)	160/92	3.541 (2.453–5.112)	<0.001	3.187 (2.179–4.661)	<0.001	3.244 (2.176–4.835)	<0.001	2.785 (1.838–4.221)	<0.001
Q4 > 143.64	252 (25.0)	218/34	13.055 (8.351–20.409)	<0.001	11.907 (7.551–18.776)	<0.001	12.067 (7.482–19.462)	<0.001	10.657 (6.492–17.493)	<0.001

*Model 1: Unadjusted OR*.

*Model 2: Adjusted for age, sex, and CTO duration*.

*Model 3: Adjusted for age, sex, CTO duration, RHR, History of T2DM, FBG, and HbA1c*.

*Model 4: Adjusted for age, sex, CTO duration, RHR, current smoker, History of T2DM, FBG, HbA1c, LVEF, and Triglycerides*.

*OR, odds ratio; CI, confidence interval; BMI, body mass index; LAP, Lipid accumulation product; CMI, Cardiometabolic Indices; WHR, waist-hip ratio; WHtR, waist-to-height ratio; BAI, body adiposity index; CVAI, Chinese visceral adiposity index*.

#### Visceral Obesity-Related Indices Were Able to Predict Poor CC

**Figure 2:**
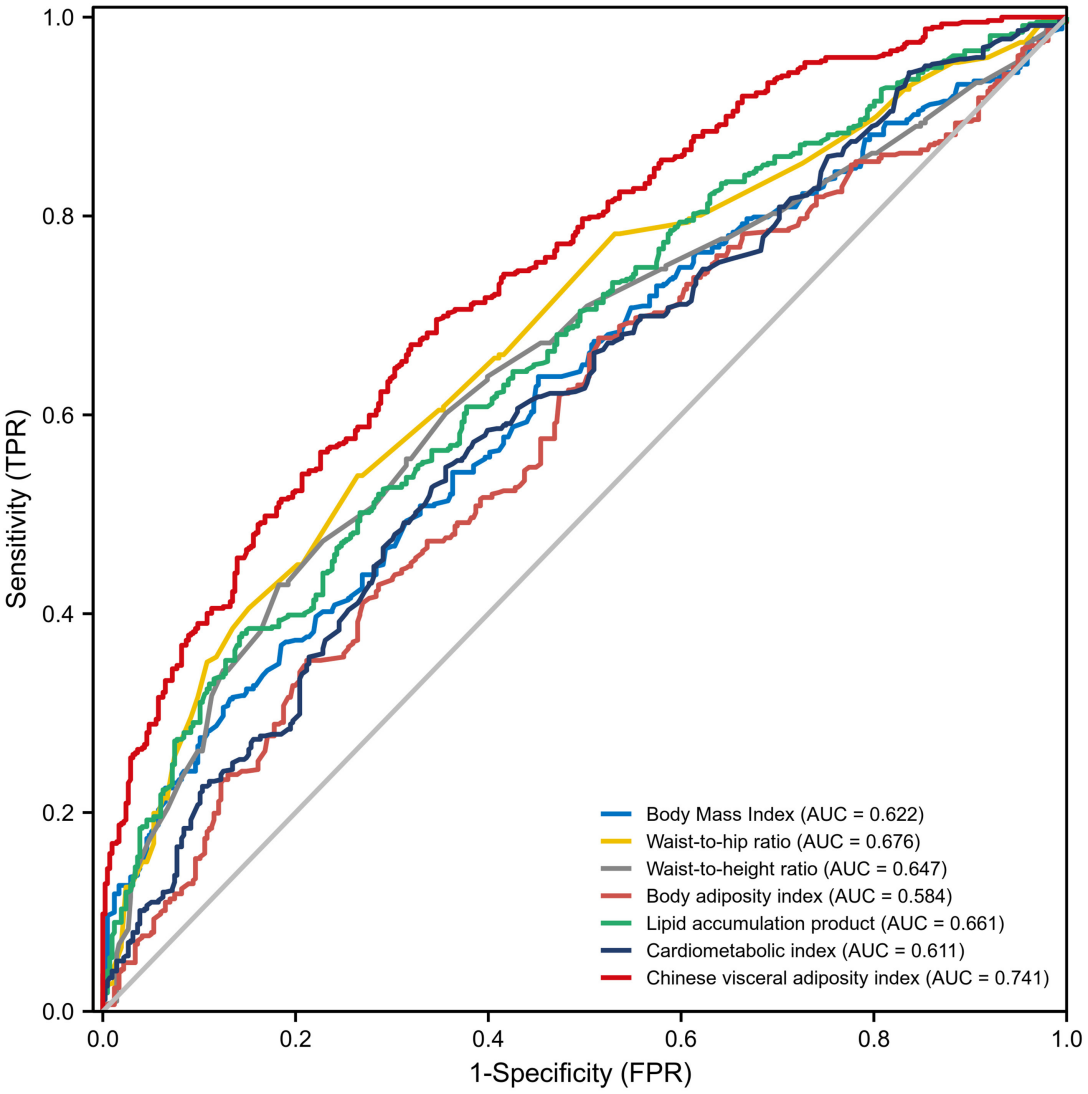
Receiver operating characteristic curves for visceral obesity-related indices for predicting the incidence of coronary collateralization. AUC, Area under curve; FPR, False positive rate; TPR, True positive rate.

According to the results of ROC analysis (Table 3; Figure 2), the AUCs for BMI, WHR, WHtR, CMI, LAP, BAI and CVAI were 0.622 (95% CI: 0.588–0.657), 0.676 (95% CI: 0.643–0.709), 0.647 (95% CI: 0.613–0.681), 0.611 (95% CI: 0.576–0.646), 0.661 (95% CI: 0.628–0.695), 0.584 (95% CI: 0.548–0.619), and 0.741 (95% CI: 0.711–0.771), respectively. CVAI exhibited the largest AUC compared with the other factors (*P*-value < 0.001). According to the *Delong* test, the predictive ability of CVAI was significantly higher than that of the other indicators, BMI, WHR, WHtR, CMI, BAI, and LAP (*Z*-value = 7.18, 3.791, 7.277, 6.539, 9.299, and 5.061, respectively) (Supplementary Table 1).

**Table 3:** Predictors of obesity-related indices for coronary collateral growth.

**Continuous variables (ROC analysis)**	**All**							
	**AUC**	**95%CI**		***P-*value**	**Best threshold**	**Specificity**	**Sensitivity**	**Youden's index**
		**Lower**	**Upper**					
Body mass index	0.622	0.588	0.657	<0.001	26.34	0.548	0.639	1.187
Waist-to-hip ratio	0.676	0.643	0.709	<0.001	0.956	0.736	0.539	1.274
Waist-to-height ratio	0.647	0.613	0.681	<0.001	0.619	0.817	0.429	1.246
Cardiometabolic index	0.611	0.576	0.646	<0.001	1.265	0.644	0.547	1.192
Lipid accumulation product	0.661	0.628	0.695	<0.001	88.865	0.856	0.38	1.236
Body adiposity index	0.584	0.548	0.619	<0.001	27.045	0.486	0.677	1.163
Chinese visceral adiposity index	0.741	0.711	0.771	<0.001	102.135	0.68	0.671	1.351

*ROC, receiver operating characteristic curve; AUC, area under the curve; CI, confidence interval*.

### Association Between CVAI and CC

#### CVAI Was an Independent Risk Factor for CC

According to baseline CVAI quartile (<71.41, 71.41–106.67, 106.67–143.64, ≥143.64), all participants were divided into four groups. The clinical characteristics of the four groups are delineated in Table 4. The 4th CVAI quartile group had higher BMI, WHR, WtHR, RHR, FBG, HbA1c, and TG levels; was more likely to have a higher prevalence of T2DM; and had a lower LVEF. Moreover, these groups had the highest incidence of MACCEs and mortality (All *P*-values < 0.001).

**Table 4:** Baseline characteristics of study population according to quartiles of CVAI.

	**Q1 (*n* = 252)**	**Q2 (*n* = 252)**	**Q3 (*n* = 252)**	**Q4 (*n* = 252)**	***P-*value**
Poor CCs	83 (32.9)	131 (52.0)	160 (63.5)	218 (86.5)	<0.001
**Rentrop**					
0	45 (17.9)	50 (19.8)	66 (26.2)	106 (42.1)	<0.001
1	79 (31.3)	87 (34.5)	75 (29.8)	84 (33.3)	
2	96 (38.1)	95 (37.7)	99 (39.3)	55 (21.8)	
3	32 (12.7)	20 (7.9)	12 (4.8)	7 (2.8)	
Age (years)	58.60 ± 9.38	61.00 ± 9.54	62.21 ± 10.55	61.73 ± 9.95	<0.001
Men	208 (82.5)	174 (69.0)	170 (67.5)	183 (72.6)	0.001
**Ethnicity (%)**					
Hans	148 (58.7)	141 (56.0)	150 (59.5)	140 (55.6)	0.553
Uyghurs	79 (31.3)	88 (34.9)	78 (31.0)	83 (32.8)	
Kazakhs	22 (8.7)	19 (7.5)	18 (7.1)	18 (7.1)	
Other ethnicities	3 (1.3)	4 (1.5)	6 (2.4)	11 (4.5)	
BMI (kg/m^2^)	25.34 ± 3.59	25.86 ± 2.55	27.89 ± 3.25	32.28 ± 5.05	<0.001
Waist-to-hip ratio	0.92 ± 0.06	0.93 ± 0.06	0.97 ± 0.07	1.01 ± 0.07	<0.001
Waist-to-height ratio	0.51 ± 0.06	0.54 ± 0.04	0.60 ± 0.06	0.66 ± 0.07	<0.001
MACCE	79 (31.3)	47 (18.7)	66 (26.2)	107 (42.5)	0.001
Death	8 (3.2)	3 (1.2)	1 (0.4)	12 (4.8)	0.005
Hypertension	143 (65.3)	161 (73.2)	146 (67)	148 (64.7)	0.313
T2DM	102 (40.5)	100 (39.7)	92 (36.5)	130 (51.6)	0.004
Current smoker	89 (35.3)	97 (38.5)	87 (34.5)	85 (33.7)	0.697
RHR (bpm)	77.06 ± 11.55	75.37 ± 10.86	77.20 ± 10.88	80.18 ± 11.10	0.002
HBP (mmHg)	128.02 ± 17.97	131.62 ± 18.99	129.82 ± 21.15	127.27 ± 17.74	0.102
SBP (mmHg)	76.44 ± 14.60	75.67 ± 12.13	74.65 ± 12.51	76.85 ± 12.11	0.955
FBG (mmol/L)	6.17 ± 2.40	6.73 ± 2.89	7.01 ± 5.30	6.41 ± 2.31	0.004
HbA1c (%)	6.80 ± 1.54	6.72 ± 1.48	6.90 ± 1.57	6.93 ± 1.47	<0.001
eGFR (ml/min/1.73 m2)	97.34 ± 16.80	96.81 ± 16.04	93.34 ± 16.25	95.66 ± 13.82	0.201
Total cholesterol (mmol/l)	3.59 ± 0.91	3.74 ± 1.00	3.86 ± 1.12	3.72 ± 1.09	0.728
Triglycerides (mmol/L)	2.03 ± 1.16	2.27 ± 1.16	2.26 ± 1.23	2.85 ± 1.52	<0.001
LDL cholesterol (mmol/l)	2.29 ± 0.85	2.32 ± 0.87	2.40 ± 0.91	2.26 ± 0.93	0.786
HDL cholesterol (mmol/l)	1.04 ± 0.30	0.99 ± 0.25	1.02 ± 0.27	0.98 ± 0.29	0.134
**Echocardiography**					
LA (mm)	36.32 ± 4.53	35.84 ± 4.19	37.10 ± 5.14	37.16 ± 5.07	<0.001
LVEDD (mm)	51.22 ± 5.43	49.79 ± 5.35	51.71 ± 6.43	54.62 ± 26.60	<0.001
LVESD (mm)	34.85 ± 5.94	33.78 ± 4.93	35.67 ± 6.89	36.73 ± 7.47	0.001
LVEF (%)	59.79 ± 7.20	60.62 ± 6.14	58.48 ± 7.99	58.11 ± 8.17	<0.001

*Data are expressed as mean ± standard deviation or number*.

*CTO, chronic total occlusion; CCs, coronary collateralizations; T2DM, type 2 diabetes mellitus; SBP, systolic blood pressure; DBP, diastolic blood pressure; FPB, fasting plasma glucose; HbA1c, glycosylated hemoglobin; HDL, high-density lipoprotein; LDL, low-density lipoprotein; LA, left atrium; RA, Right atrium; LVEDD, left ventricular end diastolic diameter; LVESD, left ventricular end systolic diameter; LVEF, left ventricular ejection fraction; ACEI, angiotensin-converting enzyme inhibitor; ARB, angiotensin receptor blocker; RHR, Resting heart rate; eGFR, estimated Glomerular filtration rate*.

**Figure 3:**
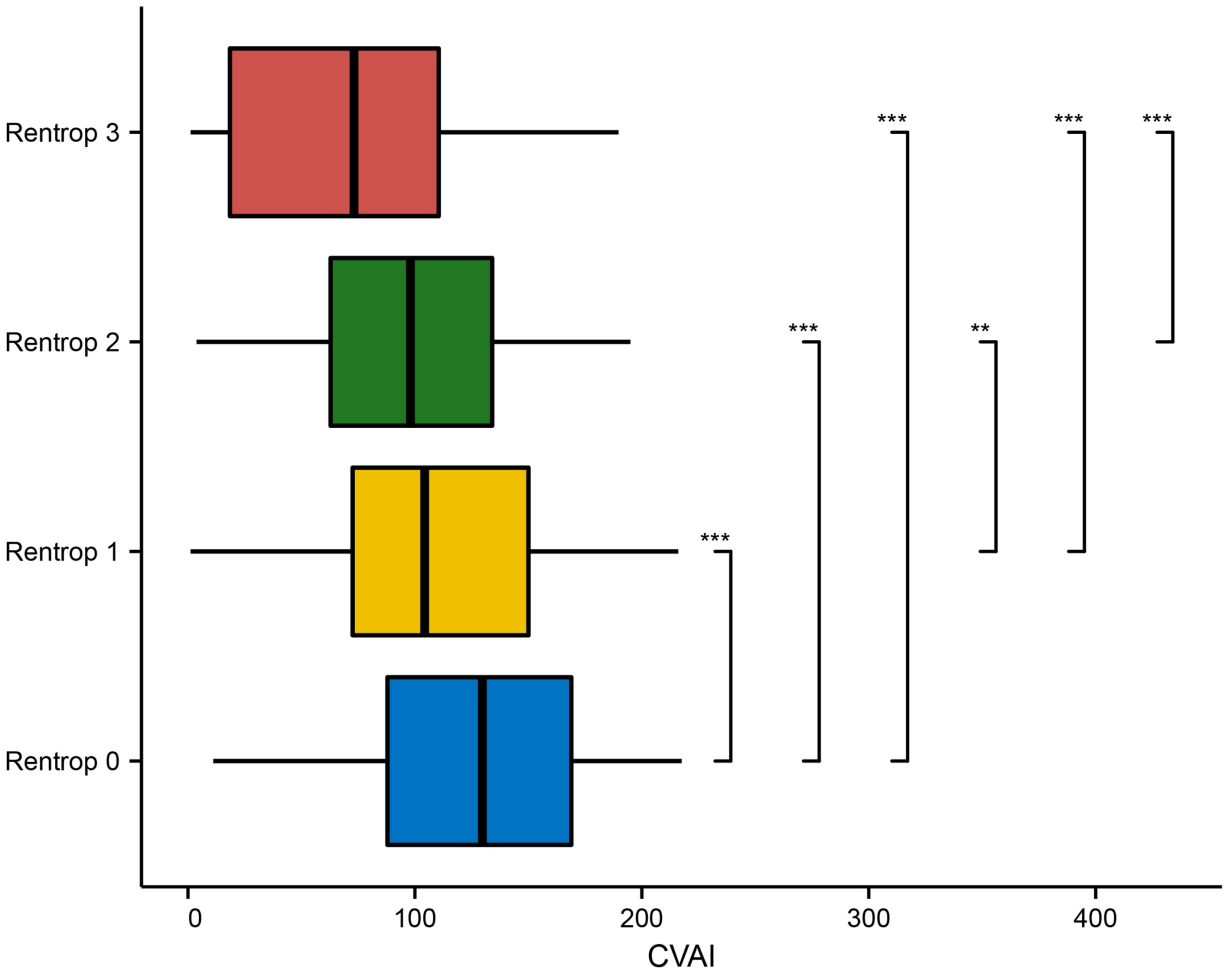
Comparison of CVAI in various Rentrop grades. CVAI, Chinese visceral adiposity index. *p < 0.05; **p < 0.01; ***p < 0.001.

As the Rentrop score of the CC level decreased, the level of CVAI increased among study subjects; at Rentrop 0 (125.846 ± 49.669), the level of CVAI was higher than that at Rentrop 1 (109.733 ± 50.889), Rentrop 2 (97.581 ± 46.820) and Rentrop 3 (72.299 ± 52.704) (*P* < 0.001; Figure 3).

Additional subgroup analyses are shown in Table 5. Patients with T2DM in higher quartiles of CVAI had a significantly increased risk of poor CC compared to those with no T2DM (adjusted OR = 9.464, 95% CI 3.832–23.373, vs. adjusted OR = 7.774, 95% CI 3.454–17.499). Female patients in the 4th CVAI quartile had a significantly higher risk of poor CC than male patients (adjusted OR = 8.347, 95% CI 1.965–35.451 vs. adjusted OR = 7.810, 95% CI 4.038–15.103). Similar results were seen in the subgroup aged >65 years old with LVEF < 60% in the 4th CVAI quartile.

**Table 5:** Subgroup analysis of the association between CVAI quartiles and coronary collateralization in patients with CTO.

	**Q1**		**Q2**			**Q3**			**Q4**		
	***N* (%)**		***N* (%)**	**Adjusted OR (95% CI)**	***P-*value**	***N* (%)**	**Adjusted OR (95% CI)**	***P-*value**	***N* (%)**	**Adjusted OR (95% CI)**	***P-*value**
**Sex**
Male	208 (82.5)	Ref.	174 (69.0)	1.882 (1.170–3.028)	0.009	170 (67.5)	1.627 (0.982–2.696)	0.059	183 (72.6)	7.810 (4.038–15.103)	<0.001
Female	44 (17.5)	Ref.	78 (31.0)	4.563 (1.738–11.980)	0.002	82 (32.5)	6.244 (2.142–18.207)	<0.001	69 (27.4)	8.347 (1.965–35.451)	<0.001
**Age**
≥65	65 (35.8)	Ref.	80 (31.7)	1.567 (0.687–3.573)	0.286	111 (44.0)	2.484 (1.092–5.651)	0.03	94 (37.3)	11.046 (3.367–36.242)	<0.001
<65	187 (68.9)	Ref.	172 (68.3)	2.725 (1.669–4.447)	<0.001	141 (56.0)	1.946 (1.123–3.372)	0.018	158 (62.7)	7.280 (3.674–14.425)	<0.001
**T2DM**
Yes	102 (40.5)	Ref.	100 (39.7)	2.336 (1.191–4.581)	0.014	92 (36.5)	1.797 (0.875–3.689)	0.11	130 (51.6)	9.464 (3.832–23.373)	<0.001
No	150 (59.5)	Ref.	152 (60.3)	2.386 (1.371–4.150)	0.002	160 (63.5)	2.421 (1.339–4.379)	0.003	122 (48.4)	7.774 (3.454–17.499)	<0.001
**LVEF**
≥60	158 (64.0)	Ref.	167 (69.0)	2.224 (1.333–3.710)	0.002	141 (58.8)	2.592 (1.478–4.545)	0.001	124 (51.0)	6.138 (2.981–12.642)	<0.001
<60	89 (36.0)	Ref.	75 (31.0)	2.536 (1.191–5.402)	0.016	99 (41.3)	1.898 (0.899–4.005)	0.093	119 (49.0)	17.785 (6.200–50.841)	<0.001

*OR, odds ratio; CI, confidence interval; BMI, body mass index; CTO, chronic total occlusion; T2DM, type 2 diabetes mellitus; HbA1c, glycosylated hemoglobin; LVEF, left ventricular ejection fraction; RHR, Resting heart rate*.

*Multiple adjustment for adjusted for age, sex, CTO duration, RHR, current smoker, History of T2DM, FBG, HbA1c, and LVEF*.

#### Increased CVAI Predicts Poor Prognosis in CTO Patients

During a mean of 11.43 ± 5.49 months of follow-up time, 299 (29.7%) patients experienced MACCEs, and 24 (2.4%) patients died [19 of them experienced cardiac death, and 5 died of respiratory failure (*n* = 2) or cerebrovascular accidents (*n* = 3)].

**Figure 4:**
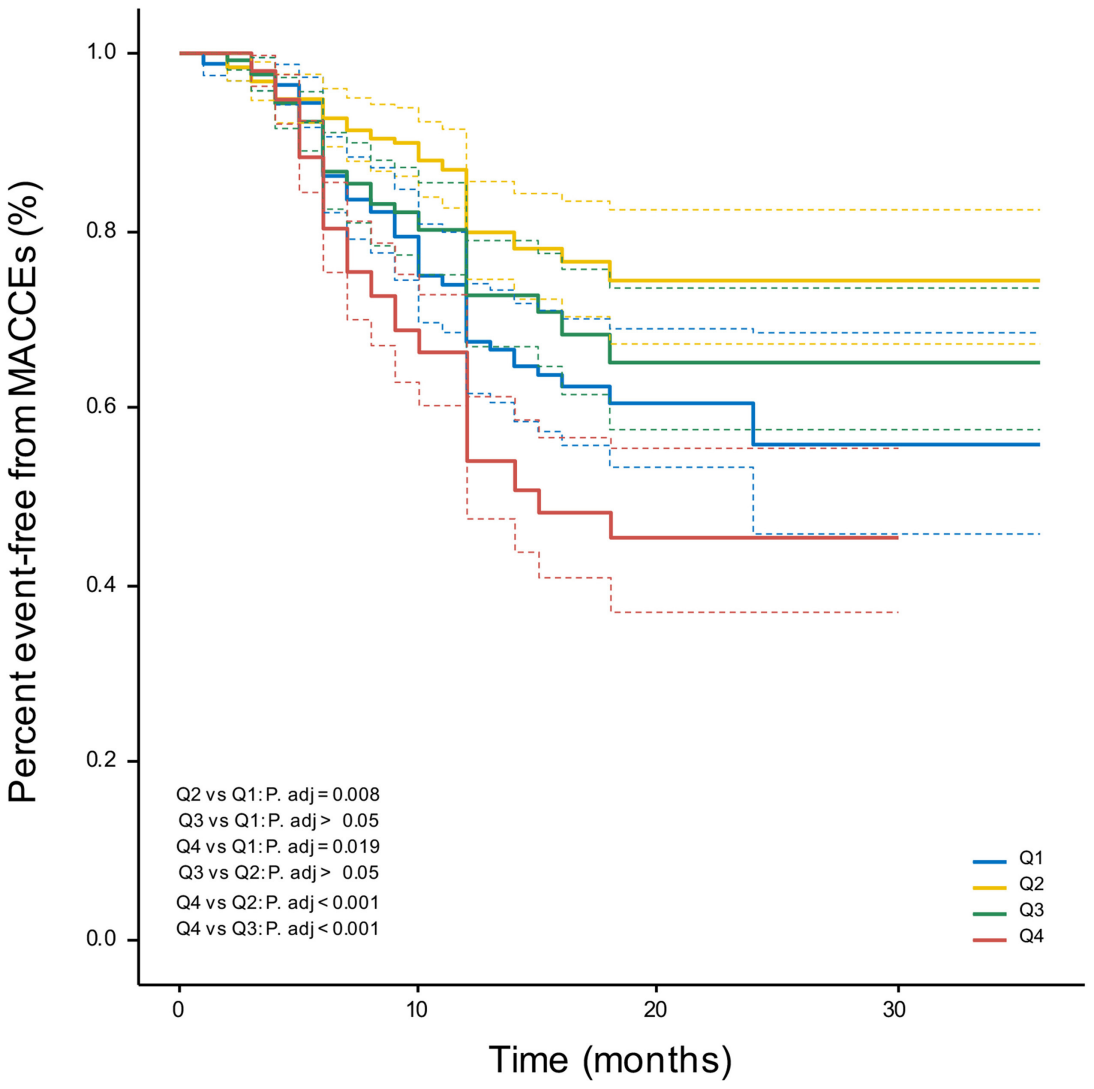
Kaplan-Meier curves. Percent event-free from MACCEs in CVAI quartile groups. MACCEs, major adverse cardio-cerebral events.

Patients in the 4th CVAI quartile had a significantly increased risk of MACCEs compared with other groups, as demonstrated by the Kaplan-Meier curves in Figure 4 (log-rank χ^2^ = 39.78, *P* < 0.001). However, compared to the 2nd CVAI quartile, the 1st CVAI quartile had a higher risk of MACCEs.

## Discussion

In this cohort study, we investigated and compared visceral obesity-related indices and their predictive value in CTO patients with poor CC. After adjusting for confounding factors, increased visceral obesity-related indices were also identified as independent risk factors for poor CC. In addition, according to ROC analysis and multivariable analyses, CVAI was the strongest predictor of increased CC, and increased CVAI predicted poor prognosis.

China has become one of the countries with the largest number of obese people, and obesity has become a major problem and major public health challenge in China. Strong evidence from prospective cohort studies has linked obesity to increased risks of CVDs and premature mortality in Chinese populations (23). Moreover, obesity has been recognized as a key risk factor for poor CC (10, 24, 25). This relationship appears to originate from disruption in adipose tissue function leading to chronic inflammation and dysregulation of the endocrine and paracrine actions of adipocyte-derived factors. These factors lead to endothelial dysfunction and impair vascular homeostasis. An altered endothelial cell phenotype and endothelial dysfunction are common among individuals with obesity and obesity-related complications (26). On the other hand, systemic proinflammation in the context of hyperglycemia, insulin resistance, oxidative stress and activation of the renin angiotensin system are systemic disturbances in obese individuals that contribute independently and synergistically (27). In brief, the pathogenesis leading to reduced formation of CC in obese individuals is regulated by multiple factors.

Emerging evidence supports that the primary culprit behind the association of obesity with cardiovascular and metabolic burden might be the pattern of fat distribution, which can be reliably quantified by imaging techniques (28). However, these imaging techniques lack economic feasibility and result in a risk of exposure to radiation. Thus, various anthropometric and cardiometabolic indices have been widely applied. In our study, the performance of BMI and WHR for estimating elevated CC was unsatisfactory compared to certain novel indices, such as CVAI, LAP, CMI, and BAI. CVAI is a novel surrogate for the assessment of metabolic health and the prediction of T2DM. BAI is a marker of adipose function and distribution, and LAP is associated with insulin resistance, reflecting central fat accumulation (29, 30). Additionally, mounting evidence supports that these visceral obesity-related indices better identify individuals at elevated risk for metabolic diseases such as T2DM and CVD (31). For instance, LAP is a better predictor of 10-year cardiovascular events than BMI, and WC and LAP are simple and accurate predictors for metabolic syndrome in undiagnosed adults (29, 32). BAI, CMI, and LAP were positively associated with hypertension risk related to variation in body fat distribution and were able to identify hypertensive participants at great risk of cardiovascular disease in the future (33, 34).

In our study, we found that CVAI exhibited the best performance in relation to coronary collateralizations among visceral obesity-related indices. Some studies have also reported that CVAI more strongly correlates with visceral fat area and is a better predictor of T2DM than BAI, BMI, and WC (35, 36). This might be because CVAI is a comprehensive index that includes age, BMI, WC, and blood lipids and hence performs better than a single index. A number of previous studies have confirmed the association of obesity and dyslipidemia with poor coronary CC (25). Compared with conventional obesity indicators, CVAI can better reflect the state of visceral fat, and we suggest that CVAI is of value as a potentially predictive biomarker for CC and the prognosis of CTO patients.

The results of survival analysis showed that patients in the 1st CVAI quartile had a higher risk of incident MACCEs than those in the 2nd and 3rd CVAI quartiles. We suggest that patients with low body weight are more susceptible to becoming ill because of poor nutritional status. In addition, some studies have reported that low body weight is correlated with worse systemic inflammation, both of which contribute to platelet aggregation and adhesion and, ultimately, stroke or systemic embolism and death (37). This finding may also be related to the single center from which the study population was recruited. Overall, multicenter studies are still required.

Visceral obesity is an independent risk factor for cardiovascular risk in the long term, and it is wise to advise patients that cardiovascular-related mortality risk can be decreased by adopting lifestyle changes such as improving nutritional habits and increasing physical activity. Personalized and appropriate physical exercise leads to important primary effects, such as restoration of endothelial function, decrease in platelet reactivity, regression or reduced progression of coronary sclerosis, and improvement of vascular repair by circulated endothelial progenitor cells (38). Overall, appropriate lifestyle management and weight loss systematically increase coronary CC.

### Study Limitations

There are several limitations in our study. First, the single-center, retrospective cohort design was an inherent limitation in this study. Second, the patient distribution was heterogeneous, as ~73% of the patients were male. Third, the Rentrop scoring system is a subjective evaluation that might be biased. The gold standard for coronary CC is the collateral flow index. Fourth, we did not consider weight changes during follow-up, and weight management might improve the prognosis of CTO patients. Finally, clinical follow-up was short, which may result in a limited number of MACCEs, particularly for hard endpoints. Therefore, we need large-scale prospective randomized studies to further determine the impact of visceral obesity-related indices and CC.

## Conclusion

In summary, this study indicated that all the increased visceral obesity-related indices were significantly associated with increased poor CC risk. After adjusting for potential risks, CVAI had the best performance for estimating CC and predicting prognosis in CTO patients.

## Data Availability Statement

The original contributions presented in the study are included in the article/Supplementary Material, further inquiries can be directed to the corresponding authors.

## Ethics Statement

The studies involving human participants were reviewed and approved by the Human Ethics Committee of the First Affiliated Hospital of Xinjiang Medical University (approval ID: K202102-17). The patients/participants provided their written informed consent to participate in this study.

## Author Contributions

Y-nY and X-ML: funding and conception and design of the study. M-JS, J-yL, and JS: data collection and processing, literature search, and data acquisition. All authors writing, critical reading, and approving the manuscript.

## Funding

This study was supported by the National Key R&D Program of China (No. 2018YFC1312804) and the Key R&D Projects in Xinjiang Uygur Autonomous Region (Nos. 2020B03002, 2020B03002-1, 2020B03002-2, 2020B03002-3). The funders had no role in the study design, data collection and analysis, preparation, interpretation, or publication of the manuscript.

## Conflict of Interest

The authors declare that the research was conducted in the absence of any commercial or financial relationships that could be construed as a potential conflict of interest.

## Publisher's Note

All claims expressed in this article are solely those of the authors and do not necessarily represent those of their affiliated organizations, or those of the publisher, the editors and the reviewers. Any product that may be evaluated in this article, or claim that may be made by its manufacturer, is not guaranteed or endorsed by the publisher.
