# Supplementary material for: Association of Visceral Obesity-Related Indices With Coronary Collateralization in Patients With Chronic Total Occlusion
Source: Front Cardiovasc Med. 2021 Oct 21;8:742855. doi: 10.3389/fcvm.2021.742855 (PMC8566720; doi:10.3389/fcvm.2021.742855)
Supplement: Supplementary file 1 [file Data_Sheet_1.docx]

| **Table supplementary 1 Pairwise comparison obesity-related indices of the AUROC in predicting coronary collateralization by the *Delong* test** | | | |
| --- | --- | --- | --- |
| Variables 1 | Variables 2 | ***Z***-statistic | ***P*** value |
| Body Mass Index | Waist-to-hip ratio | -2.639 | 0.008 |
|  | Waist-to-height ratio | -1.542 | 0.123 |
|  | Body adiposity index | 2.041 | 0.041 |
|  | Lipid accumulation product | -2.09 | 0.037 |
|  | Cardiometabolic index | 0.526 | 0.598 |
|  | Chinese visceral adiposity index | -7.18 | ＜0.001 |
| Waist-to-hip ratio | Waist-to-height ratio | 1.747 | 0.081 |
|  | Body adiposity index | 3.926 | ＜0.001 |
|  | Lipid accumulation product | 0.768 | 0.443 |
|  | Cardiometabolic index | 2.868 | 0.004 |
|  | Chinese visceral adiposity index | -3.791 | ＜0.001 |
| Waist-to-height ratio | Body adiposity index | 6.015 | ＜0.001 |
|  | Lipid accumulation product | -0.811 | 0.418 |
|  | Cardiometabolic index | 1.608 | 0.108 |
|  | Chinese visceral adiposity index | -7.277 | ＜0.001 |
| Body adiposity index | Lipid accumulation product | -3.461 | 0.001 |
|  | Cardiometabolic index | -1.126 | 0.26 |
|  | Chinese visceral adiposity index | -9.299 | ＜0.001 |
| Lipid accumulation product | Cardiometabolic index | 4.454 | ＜0.001 |
|  | Chinese visceral adiposity index | -5.061 | ＜0.001 |
| Cardiometabolic index | Chinese visceral adiposity index | -6.539 | ＜0.001 |
